# Astigmatism Associated with Allergic Conjunctivitis in Urban School Children

**DOI:** 10.1155/2019/9453872

**Published:** 2019-11-11

**Authors:** Yangho Kim, Inbo Oh, Jiho Lee, Chang Sun Sim, Yeon Suh Oh, Ju-Hyang Lee

**Affiliations:** ^1^Department of Occupational and Environmental Medicine, Ulsan University Hospital, University of Ulsan College of Medicine, Ulsan, Republic of Korea; ^2^Environmental Health Center, University of Ulsan College of Medicine, Ulsan, Republic of Korea; ^3^Department of Ophthalmology, Ulsan University Hospital, University of Ulsan College of Medicine, Ulsan, Republic of Korea

## Abstract

**Introduction:**

We first examined the association of an ocular refractive error with allergic conjunctivitis in school children and then examined this association in children attending a suburban school and an urban school.

**Methods:**

We enrolled 426 children attending a primary school in a suburban area and 550 children attending a primary school in an urban area which had a higher level of air pollution. Allergic conjunctivitis was defined as the diagnosis of this condition at any time during a child's life. The ophthalmic examinations included measurements of visual acuity and refraction, and a slit lamp examination. Skin prick tests were also performed at each school during 2018. The significance of associations was determined by the calculation of odds ratios (ORs) and 95% confidence intervals (CIs).

**Results:**

Astigmatism (increase of 1 cylindrical diopter) was associated with allergic conjunctivitis in children overall (OR = 1.287, 95% CI = 1.010 to 1.642) and in children attending the urban school (OR = 1.408, 95% CI = 1.029 to 1.926), but not in children attending the suburban school (OR = 1.040, 95% CI = 0.672 to 1.610). Allergic conjunctivitis also had a higher prevalence among children attending the urban school. The urban school had higher levels of air pollutants than the suburban school. Skin prick tests indicated that the major allergens in children with allergic conjunctivitis were house dust mites and various types of pollen.

**Conclusion:**

Astigmatism is associated with allergic conjunctivitis in children attending an urban school.

## 1. Introduction

The prevalence of allergic diseases in developed countries has increased over the past few decades, and allergies have become a major public health issue that consumes significant social expenses. Among many allergic diseases, allergic conjunctivitis is a very common ophthalmic condition that causes various ocular disorders, such as itching, burning sensations, hyperemia, and tearing on the ocular surface, that can interfere with social activities [1].

Refractive errors, such as myopia and astigmatism, are also becoming serious public health problems in school-aged children and are major causes of poor quality of life [2]. The prevalence of myopia has also increased significantly over time [3, 4]. Myopia has a high prevalence worldwide, and its incidence is 70% or more among teenagers and young adults in Asia [5]. Recent research has investigated several approaches to reduce the occurrence of myopia [6–10].

There are five types of allergic conjunctivitis, and the most common types are seasonal allergic conjunctivitis (SAC) and perennial allergic conjunctivitis (PAC). PAC lasts throughout the year, which is caused by house dust, ticks, or animal hair, and has relatively mild symptoms. SAC and PAC are mild forms of allergic conjunctivitis that are mediated by IgE. On the contrary, atopic keratoconjunctivitis (AKC), vernal keratoconjunctivitis (VKC), and giant papillary conjunctivitis (GPC) are rare types of allergic conjunctivitis that are often associated with corneal problems [11]. VKC is known to be closely associated with keratoconus (a progressive thinning and bulging of the cornea) and causes myopic astigmatism and visual disturbance [12–14]. Severe allergic conjunctivitis is rare in children, and most children with allergic conjunctivitis have SAC or PAC. However, little is known about the association of SAC and PAC with refractive errors in children, such as myopia and astigmatism.

In the present study, we first examined the association of refractive error with allergic conjunctivitis in children. Then, we compared children attending a suburban school with those attending an urban school which had a higher level of air pollution.

## 2. Materials and Methods

### 2.1. Study Area and Participants

**Figure 1:**
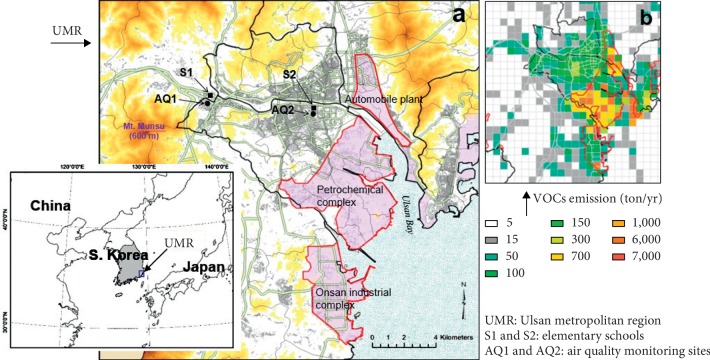
Map showing terrain in the Ulsan metropolitan region (UMR). (a) Industrial (light red shaded regions) and urbanized area (gray lines), two elementary schools (S1 and S2) for survey, and two air quality monitoring sites near each school. (b) Distribution of VOCs emissions from Korean emissions inventory data of the 2015 Clean Air Policy Support System (CAPSS) (http://airemiss.nier.go.kr/main/jsp).

The Ulsan metropolitan region (UMR) is a representative industrial city in southeastern Korea that has a population of 1.2 million. It contains a central urban area with high traffic density and large industrial complexes, including the world's largest automobile assembly plant, a shipbuilder, and a petrochemical complex along the coast (see Figure 1(a)).

Air pollution in the UMR is mainly due to emissions from urban vehicles and industrial facilities. Thus, there are higher levels of volatile organic compounds (VOCs), largely emitted from the petrochemical complex, in the industrial areas (see Figure 1(a)). The air pollution in areas close to industrial complexes is significantly higher than in other areas of the UMR, and the level of air pollution also changes seasonally due to changes in wind [17–18]

Children from two elementary schools (S1, *n* = 426; S2, *n* = 550) were recruited for this study (Figure 1(a)). Each child was in first through sixth grade. S1 is located at a central urban area near the industrial complexes, and S2 is in a suburban area that has less air pollution. All children attending the urban school lived in a central urban area, and all children attending the suburban school lived in a suburban area. Thus, people living near S1 have an increased exposure to polluted air containing industrial pollutants, such as SO_2_ and VOCs. Air quality monitoring sites (AQ1 and AQ2, Figure 1(a)) that are near each school and operated by the Korea Ministry of Environment showed that the average SO_2_ concentration during 2015 to 2017 was 31% greater at AQ1 than AQ2 (6.9 ± 3.8 *vs.* 4.7 ± 2.8 ppb).

### 2.2. Measurements

The prevalence of allergic conjunctivitis was determined by the parents' answer to the question, “Has your child ever been diagnosed with allergic conjunctivitis by a doctor?” All children in the allergic conjunctivitis group were diagnosed by a doctor at least once during their lifetimes [11].

Ophthalmic examinations (measurements of visual acuity and refraction and a slit lamp examination) and skin prick tests were performed at each school during May 2018. Examination with a portable slit-lamp was performed to determine the presence of conjunctival follicle, papilla, and injection and to identify corneal lesions. The refractive power of both eyes was measured using a Spot Vision Screener (Welch Allyn, Skaneateles Falls, NY) by trained medical assistants [17]. The spherical power, cylindrical power, and spherical equivalent were measured. The refraction power was measured at intervals of 0.25 diopter (D); myopia was indicated by (−)D and hyperopia by (+)D. The spherical equivalent was calculated as follows: (spherical D) + (½ × cylindrical D). Both eyes were measured, but only data from the right eyes were used for analysis.

The skin prick test was performed for the following allergens: *Dermatophagoides farina*, *D. pteronyssinus*, *Tyrophagus*, cockroach, ragweed, plantain, willow, mugwort, *Humulus japonicus*, alder, birch, oak, pine, *Chenopodium*, maple, dog, cat, *Alternaria*, *Cladosporium*, *Aspergillus*, shrimp, wheat flour, cow's milk, and whole egg.

Informed written consent from the parents of all participants was obtained prior to the start of the study. The study protocol and scoring procedures were approved by the Institutional Review Board of Ulsan University Hospital (IRB no. 2009-09-061).

### 2.3. Statistical Analysis

The mean values of continuous variables were compared using Student's *t*-test, and the chi square test was used to compare categorical variables (for school location and presence of allergic conjunctivitis). Then, odds ratios (ORs) and 95% confidence intervals (CIs) for allergic conjunctivitis were calculated for spherical equivalent or cylindrical D after adjustment for covariates (age, sex, parental history of allergic diseases, education level of father, conjunctival papillary hypertrophy, school location, and skin prick tests) in the logistic regression analyses. SPSS (ver. 20) was used for all statistical analyses, and a *P* value below 0.05 was considered significant.

## 3. Results

Table 1 shows the demographic characteristics of the study subjects, all of whom were primary school students enrolled in the first to sixth grade. Differences in the sex distributions of the different grades were observed in urban and total subjects, but no differences by geographical distribution in total subjects were observed.

We also characterized subjects according to the presence of allergic conjunctivitis (Table 2). There were no significant differences in the age or spherical equivalent of subjects with and without allergic conjunctivitis. However, subjects with allergic conjunctivitis had a marginally greater astigmatism (cylindrical D) than those without allergic conjunctivitis (0.63 ± 0.54 *vs.* 0.71 ± 0.63, *P*=0.051). Sex, history of asthma, low birth weight, and exposure to passive smoking were not significantly associated with allergic conjunctivitis. Subjects with allergic conjunctivitis were more likely to attend the urban school and to have a history of allergic rhinitis, atopic dermatitis, food allergy, pollen allergy, and a family history of allergic disease.

We used logistic regression analysis to calculate the ORs and 95% CIs for the relationship of allergic conjunctivitis with multiple factors using two models (Table 3). Model 1 had independent variables of astigmatism, parental history of allergic disease, education level of the father, sex, and age; Model 2 had the same independent variables as Model 1 as well as school location. Analysis of all subjects together indicated significant relationships of allergic conjunctivitis with a cylindrical D increase of 1.0 in Model 1 (OR = 1.287, 95% CI = 1.010 to 1.642) and Model 2 (OR = 1.281, 95% CI = 1.003 to 1.635). However, allergic conjunctivitis was not significantly associated with spherical equivalent (data not shown). Allergic conjunctivitis was also significantly associated with a parental history of allergic disease in Model 1 (OR = 1.778, 95% CI = 1.307 to 2.418) and in Model 2 (OR = 1.742, 95% CI = 1.279 to 2.372). Attendance at the urban school was associated with allergic conjunctivitis (OR = 1.387, 95% CI = 1.033 to 1.863). However, allergic conjunctivitis had no significant association with age, sex, or education level of the father in either model.

Children attending the urban school had a higher frequency of allergic conjunctivitis and a lower incidence of conjunctival papillary hypertrophy than those attending the suburban school (Table 4). Children at the urban school also had a higher spherical equivalent than those attending the suburban school, but the two groups had no significant differences in astigmatism (cylindrical D).

Analysis of the skin prick test results (Table 5) indicated that children with allergic conjunctivitis were more likely to test positive for *D. farinae* (52.9%), *D. pteronyssinus* (50.7%), oak (19.3%), birch (17.2%), alder (16.4%), *Tyrophagus* (15.3%), maple (11.7%), plantain (8.4%), dog (8.4%), *Chenopodium* (7.3%), *Alternaria* (6.9%), willow (6.6%), and ragweed (4.8%). In addition, children attending the urban school were more likely to have positive results than those attending suburban school.

We also used two models to separately analyze children attending the urban and suburban schools (Table 6). Model 1 had independent variables of astigmatism (cylindrical D), history of allergic disease, conjunctival papillary hypertrophy, sex, age, education level of the father, and skin prick test; Model 2 had the same independent variables as Model 1, but considered myopia (spherical equivalent) instead of astigmatism. For children attending the suburban school, both models indicated that allergic conjunctivitis was significantly associated with conjunctival papillary hypertrophy (Model 1: OR = 1.740, 95% CI = 1.081 to 2.802; Model 2: OR = 1.762, 95% CI = 1.096 to 2.835) and positive skin prick test (Model 1: OR = 1.957, 95% CI = 1.210 to 3.166; Model 2: OR = 1.918, 95% CI = 1.183 to 3.109). However, allergic conjunctivitis had no association with cylindrical diopter or spherical equivalent, or age. For children attending the urban school, allergic conjunctivitis was significantly associated with astigmatism (OR = 1.408, 95% CI = 1.029 to 1.926), parental history of allergic disease (OR = 1.668, 95% CI = 1.122 to 2.479), and positive skin prick test (OR = 1.679, 95% CI = 1.141 to 2.472) in Model 1 and with a parental history of allergic disease (OR = 1.674, 95% CI = 1.128 to 2.484) and positive skin prick test (OR = 1.723, 95% CI = 1.172 to 2.534) in Model 2. However, allergic conjunctivitis had no association with spherical equivalent.

## 4. Discussion

The mechanism responsible for the development of astigmatism is not yet clear. Outdoor activity and light exposure inhibit the development of myopia, whereas near-work activities and reading increased the risk for progression of myopia [18, 19]. However, little is known about the relationship between refractive error and allergic conjunctivitis in children [20]. To our best knowledge, this is the first study to examine the association of astigmatism with allergic conjunctivitis in children. Our statistical analysis indicated that astigmatism (increase of 1 cylindrical D) was associated with a 28.1 to 40.8% increased probability of allergic conjunctivitis.

Some previous studies have reported an association of myopia with allergic conjunctivitis [20], but the mechanism underlying this relationship has not been elucidated. Previous research also suggested that allergic conjunctivitis may occur because tear film instability (which occurs due to the change of the corneal surface associated with myopic astigmatism) causes allergen-induced substances to accumulate in the conjunctival sac, where they are adsorbed to the conjunctiva and then cause an immune reaction and inflammation [20]. However, allergic conjunctivitis (especially VKC) also causes ocular itch and irritation, resulting in habitual eye rubbing, which can induce changes of the corneal surface due to compressive and shear forces [21], and then to myopic astigmatism [12, 14, 22]. Another study found that persistent trauma to the corneal epithelium from repetitive eye rubbing or wearing of contact lenses may cause a chronic inflammatory process, in which there is a progressive loss of stromal mass and reduced biomechanical resistance, which leads to anterior corneal steepening and a decrease of the optical competence of the anterior corneal surface [23]. Many studies have documented an association of VKC with keratoconus [12, 13], and traumatic injury of the ocular surface caused by habitual rubbing could explain this relationship [21]. However, severe allergic conjunctivitis is less common in children. Most cases of allergic conjunctivitis in children are SAC and PAC, and little is known about their associations with refractive errors (myopia and astigmatism). Thus, the present study is unique in which we examined the relationship between mild allergic conjunctivitis and astigmatism in children.

However, our analysis of children attending the suburban school showed that allergic conjunctivitis was not associated with astigmatism or myopia, but was significantly associated with parental history of allergic disease, the presence of conjunctival papillary hypertrophy, and positive skin test. Allergic conjunctivitis also had a higher prevalence in children attending the urban school than the suburban school. The higher prevalence of symptomatic allergic conjunctivitis in the urban school, despite the lower incidence of conjunctival papillary changes that are characteristic of allergic conjunctivitis, may be due to the greater level of allergens. Children living in urban areas experience greater exposure to traffic-related air pollution [24]. Thus, the differences that we identified between children attending the different schools can be partly explained by differences in their exposures to air pollution [25, 26]. Previous research also reported differences in the prevalence of allergic rhino-conjunctivitis among adolescents from different cities and countries in Latin American [27] and found that residence in a rural area was significantly associated with reduced ORs for allergic rhino-conjunctivitis and asthma [28]. Another study found that urbanization was associated with childhood asthma in Hispanic Americans [29], and several studies reported associations of air pollution with asthmatic and allergic symptoms in children [25, 26]. All these findings support the presence of a higher prevalence of allergic conjunctivitis in urban areas than suburban areas, as shown in the present study.

Our study also suggests that the cause of allergic conjunctivitis in children may be sensitization to allergens, such as house dust mites and various types of pollen, in agreement with previous studies [25, 30]. Children with allergic conjunctivitis also tend to have a history of atopic dermatitis, allergic rhinitis, and food allergy, known as the “allergic march” [31, 32].

The present study has several strengths. First, we used multiple diagnostic tools in our study of factors associated with allergic conjunctivitis, including a questionnaire, an ophthalmologic examination, and skin prick tests. Second, the use of handheld refractometry (Spot Vision Screener) provides good sensitivity and specificity for identifying refractive error during mass screening [17]. Finally, we adjusted for several covariates such as demographic factors, socioeconomic status such as educational level of father, parental history of allergic diseases, and skin prick test. The association of astigmatism with allergic conjunctivitis in children attending an urban school was shown after adjustment for these covariates.

The present study also has some limitations. First, our results are based on cross-sectional analysis, and we therefore cannot infer temporal relationships or causality for any of the reported associations. Allergic conjunctivitis may occur due to myopic astigmatism, whereas allergic conjunctivitis (especially VKC) may cause ocular itch and irritation, resulting in habitual eye rubbing, followed by changes of the corneal surface (myopic astigmatism). Second, a cycloplegic test is the best method for measuring refractive errors and corneal topography for astigmatism. We used an infrared photoscreener for the measurement of refractive error because it was necessary to perform mass screening of elementary students at the school, rather than in a hospital. Use of more precise ophthalmic instruments should be used to further study the relationship of allergic conjunctivitis with astigmatism.

In conclusion, astigmatism is associated with allergic conjunctivitis in children attending an urban school, but not in children attending a suburban school. Our findings suggest this difference might be explained by the higher level of air pollution at the urban school.

## Figures and Tables

**Table 1:** Demographic characteristic of study subjects.

	Suburban			Urban			Total		
	Male	Female	*P* value	Male	Female	*P* value	Male	Female	*P* value
1^st^ grade	36 (15.9%)	31 (15.6%)	0.425	67 (23.3%)	48 (18.3%)	0.013^*∗*^	103 (20.0%)	79 (17.1%)	0.025^*∗*^
2^nd^ grade	40 (17.6%)	38 (19.1%)		51 (17.7%)	31 (11.8%)		91 (17.7%)	69 (15.0%)	
3^rd^ grade	36 (15.9%)	38 (19.1%)		53 (18.4%)	50 (19.1%)		89 (17.3%)	88 (19.1%)	
4^th^ grade	43 (18.9%)	23 (11.6%)		49 (17.0%)	39 (14.9%)		92 (17.9%)	62 (13.4%)	
5^th^ grade	44 (19.4%)	40 (20.1%)		42 (14.6%)	47 (17.9%)		86 (16.7%)	87 (18.9%)	
6^th^ grade	28 (12.3%)	29 (14.6%)		26 (9.0%)	47 (17.9%)		54 (10.5%)	76 (16.5%)	
Total	227	199		288	262		515	461	

^*∗*^ *P* value < 0.05.

**Table 2:** General characteristics of subjects with or without allergic conjunctivitis.

		Allergic conjunctivitis (−)	Allergic conjunctivitis (+)	*P* value
		*n* = 700	*n* = 273	
Age		9.2 ± 1.7	9.2 ± 1.7	0.851
Cylindrical diopter		0.63 ± 0.54 (0–4.8)	0.71 ± 0.63 (0–4.8)	0.051
Spherical equivalent		−0.60 ± 1.46 (−7.5–4.5)	−0.75 ± 1.57 (−6.0–2.5)	0.150
Sex	Male	357 (50.9%)	158 (57.7%)	0.056
	Female	345 (49.1%)	116 (42.3%)	
School location	Suburb	325 (46.3%)	101 (36.9%)	0.008
	Urban	377 (53.7%)	173 (63.1%)	
History of asthma	No	672 (95.7%)	262 (95.6%)	0.942
	Yes	30 (4.3%)	12 (4.4%)	
History of allergic rhinitis	No	466 (66.2%)	95 (34.3%)	<0.001
	Yes	237 (33.8%)	179 (65.3%)	
History of atopic dermatitis	No	546 (77.8%)	192 (70.1%)	0.012
	Yes	156 (22.2%)	82 (29.9%)	
History of food allergy	No	658 (93.7%)	234 (85.4%)	<0.001
	Yes	44 (6.3)	40 (14.6%)	
History of pollen allergy	No	680 (97.0%)	236 (86.1%)	<0.001
	Yes	21 (3.0%)	38 (13.9%)	
Father history of allergy	No	637 (91.0%)	234 (85.7%)	0.016
	Yes	63 (9.0%)	39 (14.3%)	
Mother history of allergy	No	569 (81.3%)	198 (72.8%)	0.004
	Yes	131 (18.7%)	74 (27.2%)	
Sibling history of allergy	No	639 (94.7%)	238 (90.2%)	0.012
	Yes	36 (5.3%)	26 (9.8%)	
Low birth weight	No	667 (95.2%)	262 (95.6%)	0.802
	Yes	33 (4.8%)	12 (4.4%)	
Passive smoking	No	627 (89.4%)	239 (87.2%)	0.323
	Yes	74 (10.6%)	35 (12.8%)	

**Table 3:** Odds ratios (95% CI) for having allergic conjunctivitis by astigmatism and other covariates in primary school students (*n* = 945).

	Independent variable	Odds ratios (95% CI)	*P* value
Model 1	Astigmatism (cylindrical diopter)	1.287 (1.010–1.642)	0.042
	Parental history of allergic disease (yes vs. no)	1.778 (1.307–2.418)	<0.001
	Education level of father (college vs. high school)	1.284 (0.889–1.855)	0.182
	Sex (female vs. male)	0.764 (0.571–1.021)	0.069
	Age	1.024 (0.940–1.114)	0.589

Model 2	Astigmatism (cylindrical diopter)	1.281 (1.003–1.635)	0.047
	Parental history of allergic disease (yes vs. no)	1.742 (1.279–2.372)	0.001
	Education level of father (college vs. high school)	1.255 (0.867–1.815)	0.228
	Sex (female vs. male)	0.761 (0.568–1.018)	0.066
	Age	1.028 (0.944–1.119)	0.529
	School location (urban vs suburban)	1.387 (1.033–1.863)	0.030

**Table 4:** Distribution of myopia and astigmatism in urban and suburban school.

Variables	Suburban (*n* = 426)	Urban (*n* = 550)	*P* value
Allergic conjunctivitis	101 (23.7)	173 (31.5)	0.008
Spherical equivalent	−0.52 ± 1.36	−0.74 ± 1.59	0.025
Astigmatism (cylindrical diopter)	0.65 ± 0.55	0.66 ± 0.58	0.723
Conjunctival papillary hypertrophy	152 (35.7)	112 (20.4)	<0.001

**Table 5:** Prevalence of positive skin prick tests according to allergic conjunctivitis and school location in primary school students.

	Suburban		Urban		Total	
	Conjunctivitis (−)	Conjunctivitis (+)	Conjunctivitis (−)	Conjunctivitis (+)	Conjunctivitis (−)	Conjunctivitis (+)
	(*n* = 325)	(*n* = 101)	(*n* = 377)	(*n* = 173)	(*n* = 702)	(*n* = 274)
*D. farinae*	129 (39.7%)	56 (55.4%)^*∗∗*^	142 (37.7%)	89 (51.4%)^*∗∗*^	271 (38.6%)	145 (52.9%)^*∗∗∗*^
*D. pteronyssinus*	111 (34.2%)	51 (50.5%)^*∗∗*^	123 (32.6%)	88 (50.9%)^*∗∗∗*^	234 (33.3%)	139 (50.7%)^*∗∗∗*^
*Tyrophagus*	24 (7.4%)	12 (11.9%)	35 (9.3%)	30 (17.3%)^*∗∗*^	59 (8.4%)	42 (15.3%)^*∗∗*^
Cockroach	6 (1.8%)	1 (1.0%)	11 (2.9%)	6 (3.5%)	17 (2.4%)	7 (2.6%)
Ragweed	4 (1.2%)	5 (5.0%)^*∗*^	5 (1.3%)	8 (4.6%)^*∗*^	9 (1.3%)	13 (4.9%)^*∗∗*^
Plantain	9 (2.8%)	8 (7.9%)^*∗*^	21 (5.6%)	15 (8.7%)	30 (4.3%)	23 (8.4%)^*∗*^
Willow	8 (2.5%)	6 (5.9%)	17 (4.5%)	12 (6.9%)	25 (3.6%)	18 (6.6%)^*∗*^
Mugwort	13 (4.0%)	4 (4.0%)	18 (4.8%)	11 (6.4%)	31 (4.4%)	15 (5.5%)
*Humulus japonicus*	20 (6.2%)	9 (8.9%)	10 (2.7%)	11 (6.4%)^*∗*^	30 (4.3%)	20 (7.3%)
Alder	30 (9.2%)	16 (15.8%)	30 (8.0%)	29 (16.8%)^*∗∗*^	60 (8.5%)	45 (16.4%)^*∗∗∗*^
Birch	37 (11.4%)	17 (16.8%)	39 (10.3%)	30 (17.3%)^*∗*^	76 (10.8%)	47 (17.0%)^*∗∗*^
Oak	38 (11.7%)	20 (19.8%)^*∗*^	46 (12.3%)	33 (19.1%)^*∗*^	84 (12.0%)	53 (19.3%)^*∗∗*^
Pine	6 (1.8%)	1 (1.0%)	9 (2.4%)	5 (2.9%)	15 (2.1%)	6 (2.28%)
Chenopodium	10 (3.1%)	7 (6.9%)	11 (2.9%)	13 (7.5%)^*∗*^	21 (3.0%)	20 (7.3%)^*∗∗*^
Maple	11 (3.4%)	10 (9.9%)^*∗∗*^	14 (3.78%)	22 (12.7%)^*∗∗∗*^	25 (3.6%)	32 (11.7%)^*∗∗∗*^
Dog	11 (3.4%)	4 (4.0%)	24 (6.4%)	19 (11.0%)	35 (5.0%)	23 (8.4%)^*∗*^
Cat	37 (11.4%)	13 (12.9%)	59 (15.6%)	36 (20.8%)	96 (13.7%)	49 (17.9%)
*Alternaria*	5 (1.5%)	8 (7.9%)^*∗∗*^	10 (2.7%)	11 (6.4%)^*∗*^	15 (2.1%)	19 (6.9%)^*∗∗∗*^
*Cladosporium*	5 (1.5%)	2 (2.0%)	10 (2.7%)	6 (3.5%)	15 (2.1%)	8 (2.9%)
*Aspergillus*	3 (0.9%)	1 (1.0%)	9 (2.4%)	9 (5.2%)	12 (1.7%)	10 (3.6%)
Shrimp	5 (1.5%)	4 (4.0%)	11 (2.9%)	4 (2.3%)	186 (2.3%)	8 (2.9%)
Wheat flour	2 (0.6%)	0 (0.0%)	3 (0.8%)	2 (1.2%)	5 (0.7%)	2 (0.7%)
Cow's milk	1 (0.3%)	0 (0.0%)	0 (0.0%)	1 (0.6%)	1 (0.1%)	1 (0.4%)
Egg whole	4 (1.2%)	2 (2.0%)	1 (0.3%)	2 (1.2%)	5 (0.7%)	4 (1.5%)

^*∗*^ *P* < 0.05, ^*∗∗*^*P* < 0.01, and ^*∗∗∗*^*P* < 0.001 vs. subjects without allergic conjunctivitis.

**Table 6:** Odds ratios (95% CI) for having allergic conjunctivitis by astigmatism and myopia after adjustment for covariates according to school location.

	Suburban (*n* = 420)	OR	Urban (*n* = 540)	OR
Model 1	Astigmatism (cylindrical diopter)	1.040 (0.672–1.610)	Astigmatism (cylindrical diopter)	1.408 (1.029–1.926)^*∗*^
	Parental history of allergic disease (yes vs. no)	1.666 (0.996–2.788)	Parental history of allergic disease (yes vs. no)	1.668 (1.122–2.479)^*∗*^
	Conjunctival papillary hypertrophy (yes vs. no)	1.740 (1.081–2.802)^*∗*^	Conjunctival papillary hypertrophy (yes vs. no)	1.123 (0.708–1.783)
	Sex (female vs. male)	0.662 (0.412–1.064)	Sex (female vs. male)	0.852 (0.581–1.250)
	Age	0.963 (0.835–1.112)	Age	1.048 (0.939–1.171)
	Education level of father (college vs. high school)	1.312 (0.746–2.309)	Education level of father (college vs. high school)	1.262 (0.766–2.079)
	Any skin prick test (yes vs. no)	1.957 (1.210–3.166)^*∗*^	Any skin prick test (yes vs. no)	1.679 (1.141–2.472)^*∗*^

Model 2	Myopia (spherical equivalent)	0.886 (0.739–1.062)	Myopia (spherical equivalent)	0.966 (0.852–1.095)
	Parental history of allergic disease (yes vs. no)	1.650 (0.986–2.762)	Parental history of allergic disease (yes vs. no)	1.674 (1.128–2.484)^*∗*^
	Conjunctival papillary hypertrophy (yes vs. no)	1.762 (1.096–2.835)^*∗*^	Conjunctival papillary hypertrophy (yes vs. no)	1.140 (0.720–1.805)
	Sex (female vs. male)	0.661 (0.411–1.063)	Sex (female vs. male)	0.836 (0.571–1.224)
	Age	0.927 (0.794–1.083)	Age	1.040 (0.924–1.170)
	Education level of father (college vs. high school)	1.333 (0.756–2.351)	Education level of father (college vs. high school)	1.215 (0.738–1.999)
	Any skin prick test (yes vs. no)	1.918 (1.183–3.109)^*∗*^	Any skin prick test (yes vs. no)	1.723 (1.172–2.534)^*∗*^

^*∗*^ *P* < 0.05.

## Data Availability

The data used to support the findings of this study are included within the article.
